# Correction

**DOI:** 10.1080/14686996.2023.2167688

**Published:** 2023-01-30

**Articles:**

*Interactive structural color displays of nano-architectonic 1-dimensional block copolymer photonic crystals*

Tae Hyun Park, Seunggun Yu, Jeongok Park and Cheolmin Park

https://doi.org/10.1080/14686996.2022.2156256

*Deep-learning-based inverse design of three-dimensional architected cellular materials with the target porosity and stiffness using voxelized Voronoi*

*lattices*

Xiaoyang Zheng, Ta-Te Chen, Xiaoyu Jiang, Masanobu Naito and Ikumu Watanabe

https://doi.org/10.1080/14686996.2022.2157682

*Recent advances in neuromorphic transistors for artificial perception Applications*

Wei Sheng Wang and Li Qiang Zhu

https://doi.org/10.1080/14686996.2022.2152290

**Journal**: *Science and Technology of Advanced Materials*

**Bibliometrics**: Volume 24, Number 1

When first published online, the three articles above were published with incorrect pagination from page 2 onwards. These have now been corrected accordingly, with the article ID on page one remaining unchanged, and the following pages beginning from page 2.

